# Feasibility of Telehealth Memory Assessments for a Mild Cognitive Impairment Registry: Lessons Learned from a Clinical Coordinator

**DOI:** 10.1002/alz.084022

**Published:** 2025-01-09

**Authors:** Faith Stagge, Alyssa M Lanzi, Matthew L. Cohen, Curtis L Johnson, Christopher R. Martens

**Affiliations:** ^1^ University of Delaware, Newark, DE USA

## Abstract

**Background:**

To create de novo infrastructure for ADRD research, The Delaware (DE) Center for Cognitive Aging Research (DECCAR) began a telehealth‐based mild cognitive impairment (MCI) registry. The registry included a dynamic recruitment plan and remote intake procedures to classify community‐dwelling adults as having MCI from probable Alzheimer’s Disease. The main goal of the registry was to match participants to DECCAR‐supported research studies. This work aims to report the feasibility of our telehealth methodology and observations from its development.

**Methods:**

Older adults, ages 60‐90 years old, were recruited with a variety of methods. During an initial telephone pre‐screening, individuals completed the *Modified Telephone Interview for Cognitive Status* (TICS‐m) and a questionnaire about health conditions. Individuals were invited to participate in a lengthier telehealth‐based cognitive assessment using Zoom if (1) they produced TICS‐m scores suggestive of MCI, (2) could identify a study partner, and (3) did not report non‐ADRD conditions that can cause cognitive impairment.

**Results:**

Between December, 2018, and December, 2023, n = 1029 individuals completed pre‐screening procedures (mean age was 70.1 (SD 6.56)). Paid online advertisements were the most effective recruitment source (79.30%, n = 816). Of the 1029 people who completed pre‐screening, 723 were eligible for cognitive assessment, and 571 (79%) scheduled a visit. Reasons for unsuccessful scheduling included loss of interest (n = 36), loss to follow‐up (n = 28), lack of a study partner (n = 19), and miscellaneous other reasons (n = 57). Of those who elected to schedule a telehealth assessment, 538 (94%) completed the session successfully. Telehealth assessors did not report technology to be a significant barrier. Overall, we have identified 139 individuals with MCI using this approach, which equates to 25.8% of those who successfully completed a Zoom‐based assessment.

**Conclusion:**

Telehealth platforms can be used to build an MCI research registry. In our experience, individuals who are scheduled for a telehealth assessment will generally do so successfully, perhaps because inclusion of a study partner was required. Improving the follow through from telephone pre‐screening to Zoom may require clearer advertising, removing the requirement of a study partner, or more rigorous pursuit of people who were difficult to schedule.

